# Recessive *SERPING1* Variant Leads to Kinin–Kallikrein System Control Failure in a Consanguineous Brazilian Family with Hereditary Angioedema

**DOI:** 10.3390/jcm12237299

**Published:** 2023-11-24

**Authors:** Luana Sella Motta Maia, Bettina Burger, Arije Ghannam, Fernanda Leonel Nunes, Mariana Paes Leme Ferriani, Marina Mendonça Dias, Luisa Karla Arruda, Christian Drouet, Sven Cichon

**Affiliations:** 1 Department of Biomedicine, University Hospital Basel and University of Basel, 4031 Basel, Switzerland; 2 KininX SAS, 38000 Grenoble, France; 3 Ribeirão Preto Medical School, University of São Paulo, Ribeirão Preto 14049-900, SP, Brazil; 4 Institut Cochin, INSERM UMR1016, Université Paris Cité, 75014 Paris, France; 5 Centre Hospitalier Universitaire de Grenoble, University Grenoble Alpes, 38043 Grenoble, France; 6 Institute of Medical Genetics and Pathology, University Hospital Basel, 4031 Basel, Switzerland; 7 Institute of Neuroscience and Medicine (INM-1), Research Center Juelich, 52428 Juelich, Germany

**Keywords:** hereditary angioedema, SERPING1, recessive variant, functional studies, kallikrein–kinin system, KKS control

## Abstract

**Background**: Hereditary angioedema (HAE) is a severe and potentially life-threatening disease. The most common forms are caused by variants in *SERPING1*, resulting in C1-inhibitor (C1-INH) deficiency (HAE-C1-INH). C1-INH is a serine protease inhibitor (SERPIN) that regulates multiple proteases pathways, including the kallikrein–kinin system (KKS) and its complement. In HAE-C1-INH patients, C1-INH deficiencies affect KKS control, resulting in the development of kallikrein activity in plasma and the subsequent release of bradykinin (BK). While the overwhelming majority of disease-causing *SERPING1* variants are dominant, very few recessive variants have been described. We present a large Brazilian HAE-C1-INH family with a recessive form of HAE-C1-INH. **Methods:** Blood samples of family members were investigated for protein levels of C1-INH, C4, C1q, and C1-INH function. The *SERPING1* gene was sequenced. **Results:** In two severely affected sisters, we identified a homozygous missense variant in *SERPING1* (NM_000062.3:c.964G>A;p.Val322Met). Fourteen family members were asymptomatic heterozygous carriers of the variant. Data regarding C1-INH function in the plasma showed that homozygous p.Val322Met strongly impacts C1-INH function to inhibit C1s and kallikrein (PKa). When heterozygously expressed, it affects the C1-INH control of C1s more than that of PKa. **Conclusions:** These studies of the variant’s effects on the structure–function relationship reinforce prior observations suggesting that C1-INH deficiency is a conformational disease.

## 1. Introduction

Hereditary angioedema (HAE) is a rare, potentially life-threatening disorder characterized by recurrent episodes of subcutaneous and submucosal edema, often affecting the upper airways, but also the skin and the gastrointestinal tract [1]. The worldwide prevalence of HAE has been estimated to be nearly 1/50,000 [2,3]. The most common forms of HAE (OMIM #106100), with the acronym HAE-C1-INH, are caused by variants in the *SERPING1* gene, leading to a deficiency or dysfunction of the encoded C1-inhibitor (C1-INH) [1].

C1-INH is a highly glycosylated serine protease inhibitor (SERPIN) that regulates different serine proteases via an irreversible suicide substrate mechanism [4,5]. It regulates multiple proteases pathways, including the kallikrein–kinin system (KKS), fibrinolysis, and complement. In HAE patients, C1-INH deficiency affects KKS control, resulting in the development of kallikrein activity in the plasma and the subsequent release of bradykinin (BK), the predominant mediator of enhanced vascular permeability in angioedema attacks. Two major subtypes are distinguished in HAE-C1-INH: type I (HAE-1) and type II (HAE-2). In HAE-1, the expression of the functional C1-INH protein is reduced (plasma levels and function ranging from 5% to 30% of normal), while in HAE-2, an abnormal, nonfunctional protein is synthesized [6]. The levels of C1-INH in the plasma are either normal or elevated, but its function is reduced (<50% of normal). On the one hand, C1-INH deficiency leads to reduced control of the complement [7,8], and on the other hand, it leads to kallikrein–kinin system (KKS) activation, with subsequent kininogen cleavage and accumulation of kinins on endothelial cells, including bradykinin, the most predominant mediator of vascular permeability in angioedema attacks. Complement control is impaired by C1-INH deficiency, in particular, C1 activation and lectin pathway activation, normally without any pathological consequences. However, it is followed by C4 consumption and low antigenic C4 in nearly 80% of HAE-C1-INH cases. Thus, C4 levels might be used as an HAE-C1-INH biomarker, but this is not recommended due to their reduced sensitivity. Ultimately, excessive bradykinin production leads to hyperactivation of bradykinin receptors (B1R and B2R), which then results in vasodilatation and pain [4,9]. Clinically, HAE-1 and HAE-2 are not distinguishable.

Recently, a third biochemical type of C1-INH dysfunction has been identified, called the “intermediate type” [10]. Patients with this subtype present with low antigenic C1-INH in combination with the expression of both wild-type and dysfunctional proteins. The intermediate type combines the properties of HAE types 1 and 2, in that it mimics the HAE-1 type through low antigenic C1-INH and the HAE-2 type through expression of a non-functional C1-INH variant.

HAE-C1-INH standard laboratory screening and subtype diagnosis include measurements of antigenic C1-INH and C1-INH function, as well as measurements of the C4 and C1q concentration [11,12,13]. HAE-C1-INH must be distinguished from acquired C1-INH deficiency; a rare condition resulting from increased consumption of C1-INH in vivo, with low antigenic C1q as a possible additional biomarker [14].

**Figure 1:**
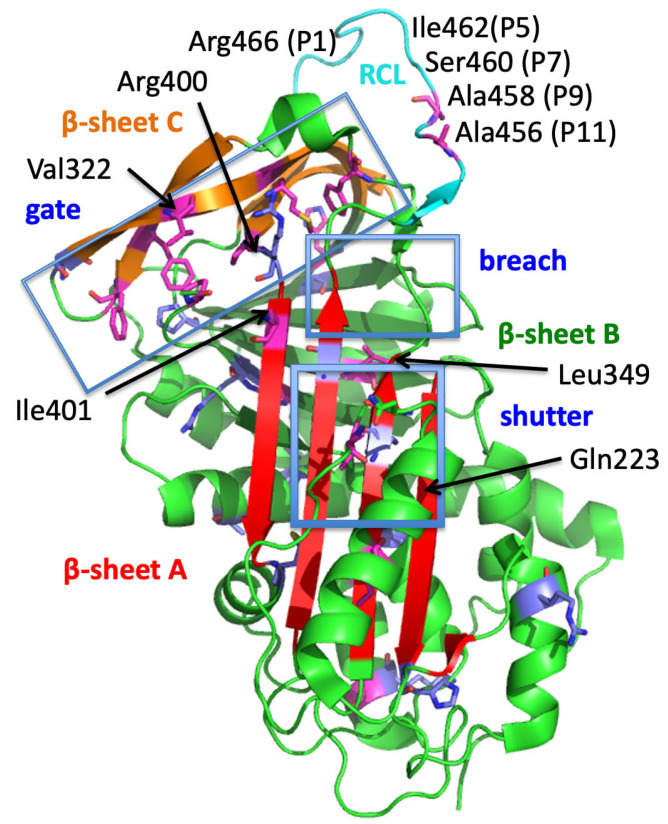
Overall structure of the native C1-INH serpin domain, with crucial regions ascribed to C1-INH serpin function. The positions of the following variants are shown: p.Val322Met, in front of the picture and in the middle of the gate; Gln_223_, which could participate in the shutter; Leu_349_, within the breach functional domain; Ile_401_, participating in the gate; and Ser_460_ and Ile_462_, as P7 and P5 of the RCL, respectively. The Arg_400_ position is protruding behind the picture. Five regions for the serpin function are presented in the 3D model of C1-INH (PDB ID 5DU3; [20]), presented using PyMOL. The model starts at Phe_122_ and lacks a significant part of the N-terminal domain (residues 1–112 of the mature protein). Strategic functional regions [21] are indicated as follows: (i) the reactive site loop (RCL) at the top of the picture, colored light blue, including Arg_466_ P1 and the hinge region (P15-P9), essential for protease recognition and RCL mobility, undergoing a conformational transformation for its insertion as strand 4A (s4A); that is, S > R transition after protease inhibition; (ii) the central β-sheet A, colored red, with the breach region located at its top, serving as a point of initial insertion of the RCL, and the shutter domain, situated close to the center of β-sheet A, which, along with the breach, assists in sheet opening and accepts the insertion of the conserved proximal hinge s4A between s3A and s5A; (iii) the gate, including s3C and s4C of β sheet C. β-sheets B and C are at the top of the model, colored green and brown, respectively. Picture drawn by Dr. Christine Gaboriaud, Grenoble.

Although all types of HAE-C1-INH are caused by variants of *SERPING1*, there are differences in terms of the localization of the variations between the different types. The variants associated with HAE-1 usually lead to the loss of one functional gene copy. These can be single-nucleotide variants or small insertions/deletions located in exonic and intronic regions (the latter usually affecting splicing), as well as larger deletions or insertions. Although one functional gene copy is still present in these patients, usually less than 50% of the functionally normal C1-INH protein remains [2,15]. The variants associated with HAE-2 usually lead to a functionally impaired protein. They are often located within the reactive centre loop (RCL), involving positions Ala_458_ (P9), Ser_460_ (P7), Ala_461_ (P6), Arg_466_ (P1), or Thr_467_ (P1′), which affect the protease target recognition by the protein and result in normal (or even higher) levels of C1-INH [3,16,17]. *SERPING1* variants associated with the intermediate form are localized within exons 3–8, with hot spots in the protein’s structural domains, mainly including its shutter, gate, and breach [10]. These structural domains refer to functional concepts that are strategic for all members of SERPIN family. SERPINs are single-chain proteins containing a conserved domain structure of 370–390 residues that interact with their target proteinase at a reactive site located within a RCL structure 30–40 amino acids from the carboxyl terminus [18]. SERPINs inhibit serine proteases through an irreversible suicide substrate mechanism when the interaction proceeds down the inhibitory arm of a branched pathway [19]. In the inhibitory pathway, the proteinase initially forms a noncovalent Michaelis-like complex through interactions with RCL residues, flanking the scissile bond P1-P1′. A metastable conformation is required for SERPIN inhibitory activity, consisting of a conserved secondary structure comprised of β-sheets A, B, and C and at least seven α-helices, from A to I. Considering only intramolecular structural changes, SERPINs can convert to the more stable latent and non-functional species. Attack of the active site serine on the RCL scissile bond leads to a covalent ester linkage between the serine of the protease active site and the backbone carbonyl of the P1 residue. The RCL, located as an exposed and flexible stretch of 15–17 residues tethered between β-sheets A and C, irreversibly inserts into the middle of β-sheet A to give an additional fully antiparallel β-sheet, with removal of the structural constraint as for a native to latent transition. The P15-P9 portion of the RCL is called the hinge region. The region of a SERPIN that controls the opening of β-sheet A and the acceptance of the RCL is the shutter domain. The region of the initial insertion of the RCL is the breach; it is located at the top of the shutter domain. The gate domain assists with this insertion in its open/closed conformations, which are associated with the loss/function of the latch interactions, respectively. The breach, shutter, and gate are the major regions that govern sheet A opening and accept the conserved hinge of the RCL in its insertion into β-sheet A [19]. Figure 1 shows the structural functional domains of C1-INH, with its RCL, breach, shutter, and gate functional regions.

More than 800 pathogenic HAE-correlated *SERPING1* variants have been reported [22], and the vast majority of them are inherited in a dominant fashion and represent missense, nonsense, or frameshift variants with a dominant negative effect [23]. Approximately 6% of the variants occur de novo [10]. In contrast with a large spectrum of dominant variants, only very few recessive forms of HAE-C1-INH have been described in a limited number of families [10,24,25,26,27,28,29,30,31]. All the recessive variants are located in exonic regions and result in HAE-1 in the majority of patients.

In the present study, we analysed the *SERPING1* gene in a large Brazilian four-generation family and identified a novel *SERPING1* variant acting in a recessive fashion. We discuss the implications of the altered protein for the manifestations of symptoms in this family.

## 2. Methods

### 2.1. Subjects

**Figure 2:**
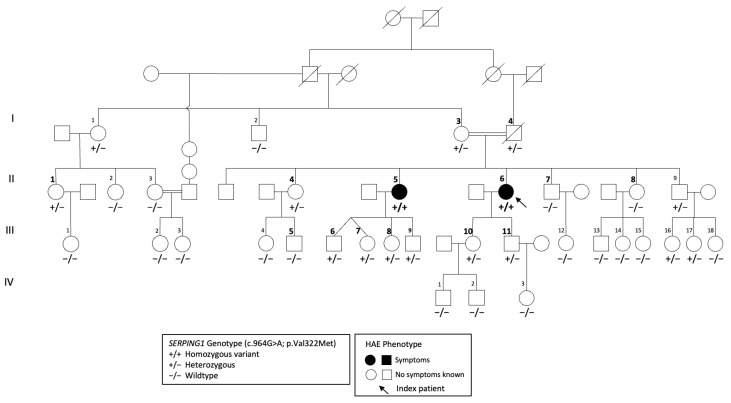
Pedigree of the family with recessive HAE-C1-INH. Homozygous individuals are marked with a solid color (+/+), and heterozygous individuals (+/−) and wild type individuals (−/−) are indicated without colors. The numbers representing biochemically examined individuals are larger and highlighted in bold.

We investigated a consanguine Brazilian four-generation family, including 23 women and 11 men aged between 1 and 81 years (Figure 2). Two sisters presented clinical symptoms of HAE and fulfilled the diagnostic criteria for HAE-1. The age of symptom onset in the affected women was 13 years (index) and 26 years (sister). Both of the sisters showed subcutaneous edema, abdominal pain, and oropharyngeal and laryngeal attacks. The index patient was diagnosed at 47 years of age. She did not have any prodrome. She had intense edema attacks occurring approximately three times a month in the subcutaneous, upper airway, and abdominal tracts. Her sister also was given the HAE late diagnosis at the age of 52 years. The symptoms were very frequent, with four episodes per month. She had prodromes 2–24 h before the edema and could correlate the edema with anxiety and stress or menses. In both sisters, the symptoms stopped after treatment with oxandrolone.

### 2.2. Severity Score

Disease severity was assessed using previously reported scores. The Bygum score focused on the age of onset, number of organs affected, and need for long-term prophylaxis [32]. The Ferraro score focused on the frequency and intensity of angioedema attacks and scaled the severity from asymptomatic to severe (mild and moderate) [33] (Table S1).

### 2.3. Levels of C1-INH, C4 and C1q

Citrate plasma samples from the individuals were prepared by centrifugating freshly collected blood at 2000× *g* for 10 min to harvest the platelet-free plasma. The samples were immediately aliquoted and stored at −80 °C until use for testing of C1-INH function.

The serum protein levels of C1-INH, C4, and C1q were measured using radial immunodiffusion. The reference intervals (5th–95th percentiles) for the C1-INH levels are between 195 and 345 mg/L (men/women), and for C4, they are between 162 and 445 mg/L (men) or 167 and 385 mg/L (women). The normal C1q levels range between 118 and 238 mg/L (men) or between 118 and 244 mg/L (women).

### 2.4. Functional Studies

Analysis of C1-INH function is the key laboratory test used to make a diagnosis of HAE-C1-INH. A C1-INH functional analysis was performed on the citrate plasma samples using chromogenic assays of the residual enzyme activity. These assays involved using a commercial kit with C1s protease (Technochrom^®^ C1-INH assay, Illkirch, France) or using kallikrein (PKa) as target, as described by Ghannam et al. [8]. Briefly, KKS was reconstituted using the purified components (FXIIa, plasma prekallikrein, and high-molecular-weight kininogen, (Enzyme research, Swansea, UK)) and was either submitted or not submitted (reference control) to incubation with a C1-INH standard (positive control) or plasma sample. The kinetics of the amidase activity, which is increased in HAE patients, were monitored using Pro-Phe-Arg-pNA (Proteogenix, Schiltigheim, France), independently of alpha2-macroglobulin control of KKS.

Functional levels are considered normal when they are ≥50% (men/women) and ≥0.61 or ≥0.42 IU/mL for men and women, respectively, using C1s protease and PKa as targets.

Spontaneous PKa activity was evaluated using the chromogenic substrate HD-Pro-Phe-Arg-*p*NA (Proteogenix, Schiltigheim, France), representing the P1-P1′ scissile bond cleaved by PKa at the C-terminus of bradykinin. It was kinetically monitored by measuring the A_405_ value at 30 °C (ThermoFisher Spectrophotometer, ThermoFisher Scientific, Waltham, Ma, USA) and expressed in nmol·min^−1^·mL^−1^. In order to determine the plasma proenzyme activation, the plasma was activated by dextran sulfate (12.5 mg·mL^−1^) at 4 °C [18], then the kinetic activity was assessed in similar conditions to the spontaneous PKa activity [34]. The reference intervals (5th–95th percentiles) are given as the spontaneous PKa activity (3.1–9.2 nmol·min^−1^·mL^−1^, men) (3.2–10.6 nmol·min^−1^·mL^−1^, women) and proenzyme PK activatability (1830–2765 nmol·min^−1^·mL^−1^, men; 1870–2985 nmol·min^−1^·mL^−1^, women).

### 2.5. Identification of C1-INH Species

The C1-INH-HAE type was characterized based on the distribution of C1-INH molecular species before and after a 15-min incubation of plasma sample in the presence or absence of target protease (1.5:1, protease:C1-INH ratio). Briefly after, both samples were submitted to 7.5% sodium dodecyl sulfate polyacrylamide gel electrophoresis (SDS-PAGE) under non-reducing conditions, followed by transfer onto an Immobilon-P^®^ polyvinylidene difluoride membrane (MilliporeSigma, Bedford, MA, USA) and submitted to anti-C1-INH immunoblotting (HRP-labeled sheep polyclonal anti-C1-INH antibody; The Binding Site, Grenoble, France). After incubation with Clarity Western ECL Blotting Substrate^®^ (BioRad, Paris, France), the bands were quantified using a ChemiDoc^®^ imaging system (BioRad) and distinguished between the 105 kDa native or latent form, the 180 kDa C1-INH-protease association, and the 95 kDa cleaved form with reference to a molecular ladder.

### 2.6. Genetic Studies

Genomic DNA was extracted from the EDTA whole blood (adults) using a DNA Wizard Genomic DNA Purification Kit (Promega, Madison, WI, USA), and from the oral mucosa (children) using a QIAamp DNA Blood Mini Kit^®^ (Qiagen, Hilden, Germany). PCR and Sanger sequencing were performed for the index patient and core family using primers for the coding region of *SERPING1*, as previously described [35]. For all other DNA, exon 6 of *SERPING1* was sequenced. The genomic DNA sequences were analyzed using the Mutation Survey or SeqMan software^®^ Version 17 (Soft Genetics LLC Lasergene; DNA Star, Inc., Madison, WI, USA). Sequence variants were identified through comparisons with GenBank accession number NG_009625.1. The significance of the variant was predicted using various tools (EVE, CADD PHRED, Mutation Taster, PolyPhen-2, and SIFT).

### 2.7. Ethical Considerations

The present study was approved by the Ethics Committee of the Clinical Hospital of Ribeirão Preto Medical School (HCRP Protocol No. 6583/2016, on 29 February 2016) and conducted according to the Helsinki guidelines. All the patients or their legal guardians provided written informed consent to participate in this study.

## 3. Results

### 3.1. Severity of Disease

The clinical severity scores of the two homozygous individuals classified them as severely affected (Bygum score: 7 and 6; Ferraro score: for both 8). Careful clinical re-examination of the heterozygous individuals excluded any HAE symptoms.

### 3.2. Biochemical Studies

The homozygous patients showed markedly reduced C1-INH function using both C1s protease and PKa as targets, with residual activities measuring less than 20% of the control capacity for C1s and less than 12.9% of the control for PKa activity (Table 1). Their serum samples also displayed a significant decrease in antigenic C1-INH and C4, with a normal antigenic C1q, excluding a diagnosis of acquired C1-INH deficiency using anti-C1-INH autoantibodies. The heterozygous patients showed a 30–50% decrease in antigenic C1-INH, associated (three individuals) or not (five individuals) with decreased antigenic C4, and a 10–40% decrease in the C1-INH control capacity of C1s; however, the control of PKa was within or very close to the normal values. The spontaneous PKa activity was increased and the proenzyme activatability was decreased in the samples from the homozygous individuals, but they were nearly normal in those from the heterozygous individuals (Figure 2 and Table 1). These data are in line with a decreased control of PKa by the p.Val322Met variant in the homozygous carriers.

### 3.3. Studies of C1-INH Molecular Species

**Figure 3:**
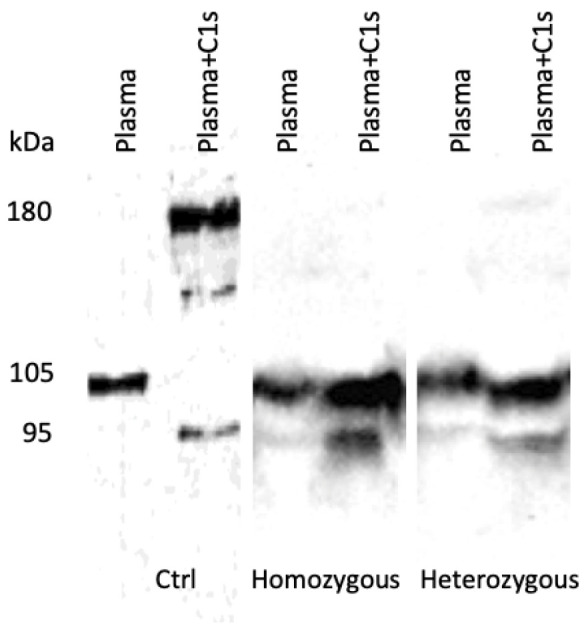
Circulating C1-INH molecular species demonstrated using an anti-C1-INH immunoblot. Plasma samples were collected from patients presenting with HAE-C1-INH, regardless of whether they were experiencing an attack or remission period. The plasma samples were incubated with C1s protease at 37 °C for 15 min and were then submitted to SDS-PAGE analysis. The 180 KDa band corresponds to the C1INH protease complex, the 105 KDa band corresponds to to native C1-INH, and the 95 KDa band corresponds to cleaved C1-INH species. Ctrl, sample from a healthy donor.

Anti-C1-INH immunoblot assays of the samples from the heterozygous individuals showed a faint band at 180 KDa, corresponding to the C1-INH-protease complex, which was undetectable in the samples from the homozygous individuals. This indicates that p.Val322Met was unable to establish a normal covalent bridge between serpin and the C1s protease. Two bands of 95 KDa and 105 KDa were displayed with comparable intensity for the samples submitted (+C1s) or not (−C1s) to the C1s protease when compared between both homozygous and heterozygous carriers. The observation of an apparent absence of serpin protease association with an abundance of both remnant 105 KDa and cleaved 95 KDa species in the (+C1s) samples indicates a low susceptibility of cleavage of p.Val322Met and quasi-impossible ester bonding with C1s, with an expression of both the wild type and variant in the heterozygous individuals (Figure 3). This profile is consistent with an expression of the p.Val322Met variant and is characteristic of an HAE-C1-INH of the intermediate type.

### 3.4. Genetic Studies

Sanger sequencing of all coding exons of the *SERPING1* gene revealed a homozygous missense variant in the index patient and in her symptomatic sister (c.964G>A,p.Val322Met) that was not listed in gnomAD. Fourteen individuals of the family were identified to be asymptomatic heterozygous carriers of the same variant, and eighteen individuals presented the wild type sequence in exon 6. No other variants were identified in the gene.

Four prediction tools (EVE score, SIFT, PolyPhen, CAAD PHRED) were used to classify the variant as pathogenic, while Mutation Taster predicted it as benign (Table S2). Prediction differences between these in silico tools were also observed for other recessive variants (i.e., c.440T>A (p.Val147Glu); c.668A>C (p.Gln223Pro); c.1202T>C (p.Ile401Thr); c.1385T>G (p.Ile462Ser)), while for two variants (c.1045C>T (p.Leu349Phe); c.1379C>T (p.Ser460Phe)), all the tools consistently predicted a pathogenic or deleterious consequence.

## 4. Discussion

In the present study, we investigated a large consanguineous Brazilian family presenting with a clinical phenotype and biological characteristics of HAE-C1-INH and identified a novel variant in the *SERPING1* gene (c.964G>A, p.Val322Met) that was recessively inherited in the HAE-C1-INH-affected individuals. This variant fits into a small series of recessive variants that are distributed across the complete *SERPING1* gene (Figure S1). Previous studies have reported eight families with homozygous or compound heterozygous probands and an unambiguous recessive form of HAE-C1-INH [10,24,25,26,27,28,29,30,31] (Table S2). The recurrent variant c.1198C>T (p.Arg400Cys), rs201363394, which was identified in five kindreds, actually appears to be a dominant variant, because several symptomatic heterozygous carriers were identified [8,36,37,38]. It has been shown that the p.Arg400Cys protein is secreted on a decreased level as an active, although quite unstable, monomer. However, it could bear a folding defect, occasionally promoting protein oligomerization and interfering with the secretion process, thus accounting for its plasma level variability. This defect is exacerbated by the nature of the mutation, since the acquired cysteine leads to the formation of non-functional homodimers through inter-molecular disulfide bonding. All the proposed phenomena could be modulated by specific environmental conditions, rendering this mutant exceptionally vulnerable to mild stress [36]. Clear evidence of c.1198C>T as a recessive variant comes from the observation of a Spanish family, with a highly symptomatic homozygous carrier, expression of the variant product in serum, and the absence of HAE-C1-INH-related symptomatology in heterozygous individuals [28].

The various prediction tools that we used to predict the pathogenic effect showed a heterogeneous picture (Table S2). This reflects the challenges in classifying the functional effects of recessive variants based on limited biological data. Interestingly, two variants affecting the same codon, p.Val322Glu and p.Val322Gly, showed a dominant inheritance pattern [10,39], and at least for one of them (p.Val322Glu), it was shown that the variant resulted in an intermediate HAE phenotype [10].

Testing of C1-INH function is the “gold standard” for the diagnosis and treatment of HAE-C1-INH. It has been established through the conventional spectrophotometric assay using C1s protease as the target, and the method is recommended for its high sensitivity for diagnostic of C1-INH deficiency [40]. However, the measurement is based on the residual activity of the C1s protease, which is not involved in the pathological process itself. For this reason, we developed an alternative enzymatic assay of C1-INH function based on KKS activation, i.e., control of PKa, which directly corresponds to the control of the pathological kinin-forming pathway. This method has been evaluated and aligned with angioedema diagnostic requirements [7,8]. The use of this method is of particular strategic importance for the investigation of the present family, where variant p.Val322Met does not similarly affect both C1s protease and KKS, as shown for the homozygous patients.

Interestingly, when we take into account the “gold standard” data from biochemical analyses of C1-INH function and antigenic C1-INH as diagnostic parameters [12,15], we would clearly expect a symptomatic phenotype in heterozygous carriers of the p.Val322Met variant. The homozygous patients presented with a low C1-INH antigenic level and function, as well as low antigenic C4, in line with HAE-C1-INH characteristics, whereas the heterozygous individuals showed subnormal to normal C1-INH parameters and normal antigenic C4. This indicates that C4 consumption did not occur above a threshold of C1s protease control by C1-INH. The heterozygous carriers of other recessive variants also displayed this feature, which could therefore be characteristic of a C1-INH deficiency associated with a recessive variant. Nevertheless, it should be emphasized that a low antigenic C4 has been shown to be inconsistent in a proportion of HAE-C1-INH patients [41]. Moreover, low antigenic C4 is not uncommon in healthy individuals; for example, those carrying one or two C4AQo or C4BQo alleles. For this reason, we do not recommend antigenic C4 for HAE-C1-INH biological testing.

Approximately 85% of patients with HAE-C1-INH are classified as HAE-1, and 15% are classified as HAE-2 [2,15]. The overlap between the elementary biochemical characteristics of HAE-1 and the intermediate type can also be seen in the present family, complicating the correct classification and potentially leading to misclassification [10]. Nonetheless, with its structure–function relationship, the identification of an intermediate type for a new variant confers an additional criterion supporting the pathogenicity in ACMG criteria [42].

Adding the analyses of C1-INH function to the control of PKa contributes to explaining the asymptomatic status of heterozygous carriers, because those values were within normal range, although they were borderline in some samples. In contrast, the homozygous individuals showed much lower PKa-correlated values, which is in accordance with homozygous individuals with other reported recessive *SERPING1* variants [22,24]. Our data indicate that the disrupting effect of C1-INH on the control of KKS, correlated with very high spontaneous PKa activity (Table 1), is of greater importance for the development of symptomatic HAE than that of the complement. Whether this is true for all carriers of a recessive variant needs to be clarified in follow-up studies. Our results clearly underline the importance of functional studies on C1-INH in combination with standard analyses on data curation to identify the pathogenicity of *SERPING1* variants. Future studies on C1-INH in other recessive families will help to understand the functional protease specificity of the protein.

In contrast to HAE-2-correlated variants that are often localized within the RCL region of the protein (70% of type 2 variants), the recessive variants do not affect this specific region of the protein, but are instead distributed across different gene regions. Val_322_ is a highly conserved position among serpins (80%), located within a buried hydrophobic environment in sheet-3C. Together with the variants at positions Phe_313_ (sheet-4C), Ser_318_ (sheet-3C), and Met_325_ (sheet-3C), Val_322_ is one of the few residues that are involved in the gate functional region. It packs against conserved gate positions at sheet-4C (Phe_313_), -3C (Met_324_), -2C (Pro_399_), and the distal hinge (Pro_498_) in native C1-INH. All these positions are also highly conserved among serpins and are affected by dominant missense pathogenic variants. Regarding the identified missense variant p.Val322Met, the Val to Met transition suggests a modified hydrophobicity and disruption of the insertion capacity of the cleaved RCL sequence into the A-sheet [10]. Subsequently, this feature explains a reduced C1-INH function and is in line with an absence of serpin–protease association, as shown in Figure 3.

The treatment of both homozygous sisters with oxandrolone improved the clinical phenotype. This effect is most likely attributable to an increase in the expression of aminopeptidase P, a metallo-peptidase of bradykinin catabolism. Aminopeptidase P levels are known to be increased in HAE-C1-INH patients submitted to danazol prophylaxis compared to patients without prophylaxis, and aminopeptidase P activity showed a significant inverse relationship to disease severity (*p* ≤ 0.001) [43].

## 5. Conclusions

The identified c.964G>A (p.Val322Met) variant exhibits a modified structure conformation with subsequent functional changes in the gate region of C1-INH. This suggests that C1-INH deficiency should be considered as a conformational disease. Biochemical analyses of standard laboratory measurements of antigenic C1-INH and antigenic C4 in asymptomatic heterozygous carriers of the recessive variant p.Val322Met would suggest a symptomatic phenotype. These individuals, however, do not show clinical symptoms. We assume that, in these individuals, the normal function of C1-INH towards the control of PKa might explain their asymptomatic status. An anti-C1-INH immunoblot analysis showed that p.Val322Met is not capable of establishing a covalent association between serpin and C1s protease, in accordance with the hypothesis of a low accessibility of p.Val322Met to target proteases and a disrupted function of the gate domain.

Data of further asymptomatic heterozygous *SERPING1* variant carriers could improve our knowledge of strategic amino acids that are crucial for particular functional domains of C1-INH, i.e., gate, breach, and shutter.

## Figures and Tables

**Table 1:** Laboratory characteristics of the HAE patients and their relatives. Whereas the values for antigenic C1-INH, C1-INH function, and antigenic C4 are below normal for homozygous and heterozygous carriers of the c.964G>A, p.Val322Met variant, this only applies for the homozygous carrier (II.3; II.4) in case of the PKa activity. Arrows indicate values below the 5th quantile (↓) or above the 95th quantile (↑), which are indicated in bold. Reference intervals are given below the table.

Pedigree Position	Sex	Age	Genetics	Antigenic C1-INH (mg/L) *	C1-INH Function—C1s *	Antigenic C4 (mg/L) *	Antigenic C1q (mg/L) *	Kallikrein (PKa) Activity			
								Spontaneous PKa Activity (nmol/mL/min) *	Proenzyme Activatability (nmol/mL/min) *	C1-INH Function—PKa (IU/mL) *	C1-INH Function—PKa (%)
II.6	F	50	Homozygous	**40** **↓**	**17%↓**	**64.4↓**	191	**124↑**	**1601↓**	**<0.11↓**	**<12.9↓**
II.5	F	52	Homozygous	**95** **↓**	**16%** **↓**	**64.4** **↓**	230	**193.6** **↑**	**1769** **↓**	**<0.11** **↓**	**<12.9** **↓**
III.6	M	26	Heterozygous	**181** **↓**	**32%** **↓**	**161** **↓**	Not assessed	**56.2** **↑**	2203	**0.36** **↓**	42
I.3	F	75	Heterozygous	**169** **↓**	85%	493	Not assessed	**42** **↑**	1997	0.76	102
III.7	F	26	Heterozygous	**164** **↓**	**45%** **↓**	171	Not assessed	**20.5** **↑**	**1842** **↓**	0.45	61
III.8	F	21	Heterozygous	**164** **↓**	**23%** **↓**	**64** **↓**	Not assessed	**12.6** **↑**	**1648** **↓**	**0.29** **↓**	39
II.4	F	54	Heterozygous	**128** **↓**	**41%** **↓**	279	Not assessed	**12.6** **↑**	**1830** **↓**	**0.41** **↓**	55
III.11	M	28	Heterozygous	**189** **↓**	**29%** **↓**	242	Not assessed	**12.2** **↑**	1857	**0.59** **↓**	69
III.10	F	31	Heterozygous	**128** **↓**	**10%** **↓**	**161** **↓**	Not assessed	10.6	2101	**0.39** **↓**	52
II.1	F	42	Heterozygous	**186** **↓**	**29%** **↓**	346	Not assessed	8.5	1889	0.61	109
II.8	F	46	Wild type	265	Not assessed	632	Not assessed	7.2	1936	1.36	183
III.5	M	25	Wild type	164	Not assessed	306	Not assessed	6.6	1872	1.49	175
II.7	M	48	Wild type	450	Not assessed	217	Not assessed	8.5	1899	1.27	149

* Reference intervals: antigenic C1-INH (mg/L): 195–345 (men/women); C1-INH function—C1s: ≥50% (men/women); antigenic C4 (mg/L): 162–445 (men); 167–385 (women); antigenic C1q (mg/L): 118–238 (men); 118–244 (women); spontaneous PKa activity (nmol·min^−1^·mL^−1^): 3.1–9.2 (men); 3.2–10.6 (women); proenzyme activatability (nmol·min^−1^·mL^−1^): 1830–2765 (men); 1870–2985 (women); C1-INH function—PKa (IU/mL): ≥0.61 (men); ≥0.42 (women).

## Data Availability

A repository for the novel variant is accessible. According to the Author Guidelines, the novel variant c.964G>A; p.(Val322Met) has been successfully introduced into the Global Variome shared LOVD (https://databases.lovd.nl/shared/genes/SERPING1), accessed on 22 November 2023.

## Supplementary Materials

The following supporting information can be downloaded at: https://www.mdpi.com/article/10.3390/jcm12237299/s1, Table S1 Clinical severity score. Assignment of the Bygum score based on the age at disease onset, number of organs ever affected and need for long-term prophylaxis. Table S2. Variants in the SERPING1 gene identified in homozygous probands as well as two dominant variants that affect the codon 322 but result in a different amino acid substitution. Figure S1. Distribution of the reported recessive variants in the SERPING1 gene according to their mature protein numbering. The reference [8,10,24,25,26,27,28,29,30,31,32,33,36,37,38,39,44,45,46,47] are cited in Supplementary Materials.
