# Supplementary material for: Recessive SERPING1 Variant Leads to Kinin–Kallikrein System Control Failure in a Consanguineous Brazilian Family with Hereditary Angioedema
Source: J Clin Med. 2023 Nov 24;12(23):7299. doi: 10.3390/jcm12237299 (PMC10707408; doi:10.3390/jcm12237299)
Supplement: Supplementary file 1 [file jcm-12-07299-s001.zip › jcm-2720525-supplementary.pdf]

Table S1 Clinical severity score. Assignment of the Bygum score based on the age at disease onset, number of organs ever affected, and need for long-term prophylaxis (1). Clinical severity score cumulated, 0–10 points. The Ferraro classification included the frequency of symptoms and their intensity based on averaged data since disease onset (for patients with long-term histories, the mean values for the past five years were taken into account) (2). * The sum of the frequency and intensity scores was used to classify the severity of the disease as follows: severe (≥7 points), moderate (5 or 6 points), mild (≤4 points), and asymptomatic (0 points).

	Bygum points	Ferraro points*
Age at onset: 0–5 years	3	n/a
Age at onset: 6–10 years	2	n/a
Age at onset: 11–20 years	1	n/a
Age at onset: >20 years	0	n/a
Skin edema	1	n/a
Painful abdominal edema	2	n/a
Laryngeal edema	2	n/a
Other clinical manifestations	1	n/a
Long-term prophylaxis	1	n/a
Frequency: >one episode a month	n/a	3
Frequency: between 6 and 11 episodes a year	n/a	2
Frequency: <6 episodes a year	n/a	1
no symptoms of angioedema	n/a	0
Intensity: presence of discomfort but no disruption in daily activity	n/a	2
Intensity: discomfort reducing normal daily activity	n/a	4
Intensity: inability to work or perform daily activity and/or necessity of hospital care	n/a	5

Table S2. Variants in the *SERPING1* gene identified in homozygous probands as well as two dominant variants that affect codon 322 but result in a different amino acid substitution.

Region	DNA change	Predicted protein change (immature)	C1-INH domain	Predictive algorithm: EVE Score[1]	SIFT[2]	Poly Phen2[3]	CADD PHRED[4]	Mutation Taster[5]	MAF[6]	Existing variant	Reference
exon 1	c.-161A>G	5'UTR		-	-	-	20.7	-	n.d.	rs1291031675	(3, 4)
exon 3	c.440T>A	p.Val147Glu	helix A'	*0.262*	0.01	0.458	16.53	Benign	n.d.	n.d.	(5)
exon 4	c.668A>C	p.Gln223Pro	s2A	0.823	0.01	0.694	25.1	Deleterious	n.d.	n.d.	(6)
exon 6	c.964G>A	p.Val322Met	s3C	0.689	0.00	0.99	26.7	Benign	n.d.	n.d.	present study
exon 6	c.965T>G‡	p.Val322Gly	s3C	0.562	0.00	0.996	24.7	Benign	n.d.	n.d.	(7)
exon 6	c.965T>A‡	p.Val322Glu	s3C	0.793	0.00	0.973	24.7	Benign	n.d.	n.d.	(6)
exon 6	c.965_967delTGC‡	p.Val322_323delinsAla	s3C	-	-	-	-	Deleterious	n.d.	n.d.	(6)
exon 7	c.1045C>T	p.Leu349Phe	s2B	0.548	0.03	*0.234*	23.4	Deleterious	6.36E-05	rs141075266	(6, 8)
exon 7	c.1198C>T⊥	p.Arg400Cys	loop s2C/s6A	0.708	0.00	0.99	23.0	Deleterious	4.77E-05	rs201363394	(9-13)
exon 7	c.1202T>C	p.Ile401Thr	s6A gate	0.535	0.00	0.909	24.7	Deleterious	n.d.	rs1263371770	(14, 15)
exon 8	c.1379C>T⊥	p.Ser460Phe	RCL[7] P7	0.658	0.00	0.993	22.8	Deleterious	n.d.	n.d.	(14, 15)
exon 8	c.1385T>G	p.Ile462Ser	RCL[7] P5	0.394	0.00	*0.029*	17.2	Deleterious	n.d.	rs763451792	(16)

[1]EVE (evolutionary model of variant effect) is an algorithm predicting the clinical significance of human variants based on sequences of divers organism across evolution (17). Score ranges from 1, most pathogenic, to 0, most benign. EVE class variants are categorized as pathogenic (underlined), uncertain (black), or benign (italics). No prediction possible is indicated with a minus.

[2]SIFT (Sorting Intolerant from Tolerant) is an algorithm predicting the effect of amino acid substitutions on protein function, with their negative results being more trusty than the positive ones (18). Scores <0.05 suggesting a deleterious change are underlined.

[3]PolyPhen-2 (polymorphism phenotyping 2) is a tool that predicts the possible impact of an amino acid substitution on the structure and function of a human protein (19). Protein transcript ENST00000278407 was used for the calculation. Score values <0.700 indicate a benign variant (shown in italics), including those with the variability of a possibly damaging effect (black). Likely damaging variants are underlined.

[4]CADD (combined annotation dependent depletion) is a tool that integrates diverse genome annotations to score the deleteriousness of single nucleotide variants as well as insertion/deletions variants in the human genome (20). The PHRED-like scaled CAAD score simplifies the interpretation of the raw CAAD score by

ranking it relative to all possible substitutions in the human reference genome (e.g., a score of 20 or greater indicates a raw score in the top 1% of all possible reference genome SNVs). The higher the score, the higher the probability of a deleterious effect of a variant.

[5]MutationTaster2021 is a tool predicting the effects of DNA variants, integrating publicly available sources of data, e.g., gnomAD, ExAC pLI scores, and ClinVar. It provides a binary prediction (deleterious or benign).

[6]Data of the minor allele frequency (MAF) of a variant originates from gnomADv2; variants not listed are indicated as n.d.

[7]RCL (reactive center loop), a sequence motif that is specifically recognized by target proteases.

‡This dominant variant affects the codon present in homozygous carriers but results in a different amino acid substitution.

⊥This variant has been described as likely pathogenic in several heterozygous carriers.

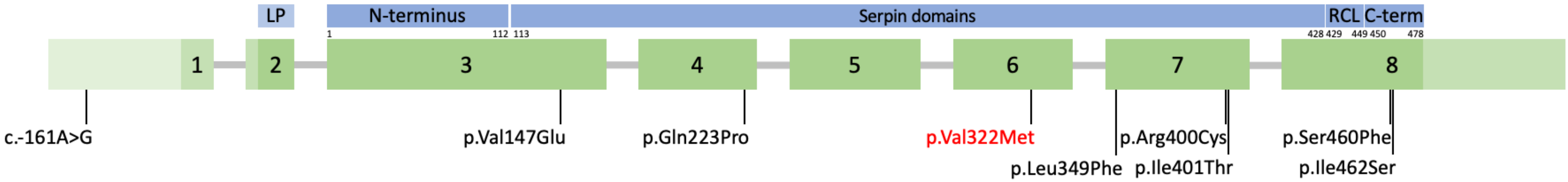

Figure S1. Distribution of the reported recessive variants in the *SERPING1* gene according to their mature protein numbering. The present variant is highlighted in red. Green boxes represent exons 1–8 with the CDS in dark green, light green boxes represent untranslated regions (UTRs). The main protein domains are depicted in blue. RCL, reactive centre loop; LP, leader peptide; serpin, serine protease inhibitor.

Supplemental References

1. Bygum A, Fagerberg CR, Ponard D, Monnier N, Lunardi J, Drouet C. Mutational spectrum and phenotypes in Danish families with hereditary angioedema because of C1 inhibitor deficiency. Allergy. 2011;66(1):76-84.doi:10.1111/j.1398-9995.2010.02456.x.
2. Ferraro MF, Moreno AS, Castelli EC, Donadi EA, Palma MS, Arcuri HA, et al. A single nucleotide deletion at the C1 inhibitor gene as the cause of hereditary angioedema: insights from a Brazilian family. Allergy. 2011;66(10):1384-90.doi:10.1111/j.1398-9995.2011.02658.x.
3. Büyüköztürk S, Eroglu BK, Gelincik A, Uzümcü A, Özseker F, Colakoglu B, et al. A Turkish family with a novel mutation in the promoter region of the C1 inhibitor gene. J Allergy Clin Immunol. 2009;123(4):962-4.doi:10.1016/j.jaci.2008.12.022.
4. Kesim B, Uyguner ZO, Gelincik A, Mete Gökmen N, Sin AZ, Karakaya G, et al. The Turkish Hereditary Angioedema Pilot Study (TURHAPS): the first Turkish series of hereditary angioedema. Int Arch Allergy Immunol. 2011;156(4):443-50.doi:10.1159/000323915.
5. Nabilou S, Pak F, Alizadeh Z, Fazlollahi MR, Houshmand M, Ayazi M, et al. Genetic study of hereditary angioedema type I and type II (first report from Iranian patients: describing three new mutations). Immunol Invest. 2022;51(1):170-81.doi:10.1080/08820139.2020.1817068.
6. Ponard D, Gaboriaud C, Charignon D, Ghannam A, Wagenaar-Bos IGA, Roem D, et al. SERPING1 mutation update: Mutation spectrum and C1 inhibitor phenotypes. Hum Mutat. 2020;41(1):38-57.doi:10.1002/humu.23917.
7. Hashimura C, Kiyohara C, Fukushi JI, Hirose T, Ohsawa I, Tahira T, et al. Clinical and genetic features of hereditary angioedema with and without C1-inhibitor (C1-INH) deficiency in Japan. Allergy. 2021;76(11):3529-34.doi:10.1111/all.15034.
8. Ren Z, Zhao S, Li T, Wedner HJ, Atkinson JP. Insights into the pathogenesis of hereditary angioedema using genetic sequencing and recombinant protein expression analyses. J Allergy Clin Immunol. 2023;151(4):1040-9 e5.doi:10.1016/j.jaci.2022.11.027.
9. López-Lera A, Favier B, de la Cruz RM, Garrido S, Drouet C, López-Trascasa M. A new case of homozygous C1-inhibitor deficiency suggests a role for Arg378 in the control of kinin pathway activation. J Allergy Clin Immunol. 2010;126(6):1307-10 e3.doi:10.1016/j.jaci.2010.07.037.
10. Caccia S, Suffritti C, Carzaniga T, Berardelli R, Berra S, Martorana V, et al. Intermittent C1-Inhibitor Deficiency Associated with Recessive Inheritance: Functional and Structural Insight. Sci Rep. 2018;8(1):977.doi:10.1038/s41598-017-16667-w.
11. Pappalardo E, Caccia S, Suffritti C, Tordai A, Zingale LC, Cicardi M. Mutation screening of C1 inhibitor gene in 108 unrelated families with hereditary angioedema: functional and structural correlates. Mol Immunol. 2008;45(13):3536-44.doi:10.1016/j.molimm.2008.05.007.
12. Ghannam A, Sellier P, Defendi F, Favier B, Charignon D, Lopez-Lera A, et al. C1 inhibitor function using contact-phase proteases as target: evaluation of an innovative assay. Allergy. 2015;70(9):1103-11.doi:10.1111/all.12657.
13. Gosswein T, Kocot A, Emmert G, Kreuz W, Martinez-Saguer I, Aygoren-Pursun E, et al. Mutational spectrum of the C1INH (SERPING1) gene in patients with hereditary angioedema. Cytogenet Genome Res. 2008;121(3-4):181-8.doi:10.1159/000138883.
14. Mete Gökmen N, Gülbahar O, Onay H, Peker Koc Z, Özgül S, Köse T, et al. Deletions in SERPING1 lead to lower C1 Inhibitor function: lower C1 inhibitor function can predict disease severity. Int Arch Allergy Immunol. 2019;178(1):50-9.doi:10.1159/000492583.
15. Mete Gökmen N, Rodriguez-Alcalde C, Gülbahar O, López-Trascasa M, Onay H, López-Lera A. Novel homozygous variants in the SERPING1 gene in two Turkish families with hereditary angioedema of recessive inheritance. Immunol Cell Biol. 2020;98(8):693-9.doi:10.1111/imcb.12362.

16. Blanch A, Roche O, Urrutia I, Gamboa P, Fontán G, López-Trascasa M. First case of homozygous C1 inhibitor deficiency. J Allergy Clin Immunol. 2006;118(6):1330-5.doi:10.1016/j.jaci.2006.07.035.
17. Frazer J, Notin P, Dias M, Gomez A, Min JK, Brock K, et al. Disease variant prediction with deep generative models of evolutionary data. Nature. 2021;599(7883):91-5.doi:10.1038/s41586-021-04043-8.
18. Sim NL, Kumar P, Hu J, Henikoff S, Schneider G, Ng PC. SIFT web server: predicting effects of amino acid substitutions on proteins. Nucleic Acids Res. 2012;40(Web Server issue):W452-7.doi:10.1093/nar/gks539.
19. Adzhubei IA, Schmidt S, Peshkin L, Ramensky VE, Gerasimova A, Bork P, et al. A method and server for predicting damaging missense mutations. Nat Methods. 2010;7(4):248-9.doi:10.1038/nmeth0410-248.
20. Rentzsch P, Witten D, Cooper GM, Shendure J, Kircher M. CADD: predicting the deleteriousness of variants throughout the human genome. Nucleic Acids Res. 2019;47(D1):D886-D94.doi:10.1093/nar/gky1016.
